# Radiation exposure in transradial versus transfemoral access in endovascular treatment of intracranial aneurysms

**DOI:** 10.1007/s00330-026-12603-7

**Published:** 2026-05-04

**Authors:** Gregor Peter, Christian Thaler, Luca Meucci, Felix Schlicht, Matthias Bechstein, Vincent Geest, Helge Kniep, Maxim Bester, Jens Fiehler, Lukas Meyer

**Affiliations:** Department of Diagnostic and Interventional Neuroradiology, University Medical Center Hamburg-Eppendorf, Hamburg, Germany

**Keywords:** Radiation exposure, Endovascular aneurysm treatment, Access route, Dose area product

## Abstract

**Objectives:**

To compare radiation exposure between transradial (TRA) and transfemoral (TFA) access and identify treatment-related factors associated with radiation exposure in endovascular treatment of intracranial aneurysms.

**Materials and methods:**

This retrospective single-center study analyzed consecutively treated patients receiving endovascular aneurysm treatment (EAT) from May 2023 to April 2025 at the University Medical Center Hamburg-Eppendorf. EAT was performed by nine experienced board-certified neuroradiologists. The primary outcome was the radiation exposure defined as dose area product (DAP) (Gy·cm^2^). In addition to access route (TRA vs. TFA), patient-related (e.g. age, gender, aneurysm location) and procedure-related (e.g., interventionalist, incidental vs. symptomatic, used device) characteristics were analyzed with regard to radiation exposure using uni- and multivariable linear regression analysis.

**Results:**

A total of 209 patients (156 female, 53 male; median age 59 [51–68]) were analyzed. Median DAP was 72.2 (51.6–96.7). Multivariable linear regression analyses revealed that radiation exposure did not differ significantly between TRA and TFA (*β* = 8.13 [−3.79 to 20.01]; *p* = 0.18). Stent-assisted coiling (*β* = 55.82 [34.91–76.74]; *p* < .001) and female gender (*β* = −14.95 [−27.5 to −22.41]; *p* = 0.02) were associated with increased DAP. Further patient- and procedure-related variables were not significantly associated with radiation exposure.

**Conclusion:**

In this study, the choice between TRA and TFA had no significant impact on radiation exposure during endovascular aneurysm treatment. Stent-assisted coiling was independently associated with increased radiation exposure during endovascular treatment of intracranial aneurysms.

**Key Points:**

***Question*** *Understanding and minimizing radiation exposure in endovascular aneurysm treatment is essential, as the increasing use of transradial access necessitates comparison with the established transfemoral route.*

***Findings*** *Radiation exposure did not differ between transradial and transfemoral access, while higher doses were independently associated with stent-assisted coiling procedures of increased complexity.*

***Clinical relevance*** *Our findings support the safety of transradial access regarding radiation exposure, allowing operators to select the most suitable access route based on patient anatomy and preference without increasing radiation risk.*

**Graphical Abstract:**

**Figure:**
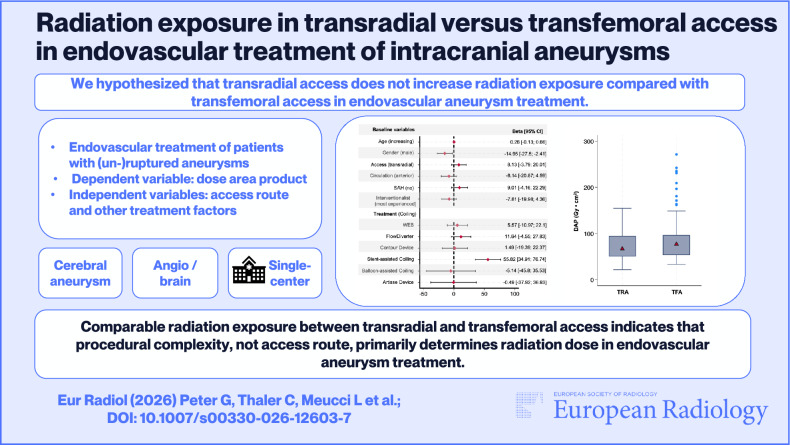

## Introduction

Endovascular aneurysm treatment (EAT) is considered for the majority of patients with cerebral aneurysms [1]. With advances in catheter and device technology, alternative vascular access routes have gained importance. As endovascular techniques have advanced, alternative vascular access routes have also emerged. While the transfemoral approach (TFA) has traditionally served as the standard access route, the transradial approach (TRA) is increasingly utilized in neurointerventional procedures [2–4].

Reports of potential benefits associated with TRA include improved patient comfort, earlier mobilization, and reduced access-site complications [2–5]. In addition, several studies have already observed the safety and efficacy of TRA in neuroendovascular procedures [5–10]. However, several procedural characteristics of TRA may theoretically increase radiation exposure compared with TFA. Catheter navigation from the radial artery to the intracranial circulation can be more complex, particularly in the presence of anatomical variants and vessel tortuosity (e.g. radial loops, subclavian and aortic arch elongation). The smaller vessel caliber may also limit catheter support and stability, potentially requiring more repositioning and additional angiographic acquisitions. These technical factors may be more relevant in less experienced operators or in early institutional adoption, which could translate into longer fluoroscopy time (FT) and a higher number of angiographic series.

Most patients diagnosed with cerebral aneurysms are relatively young, with a reported mean age at diagnosis ranging from 43 to 55 years [11, 12]. Considering the comparatively early age of onset and generally favorable life expectancy, radiation safety becomes a particularly important aspect of long-term care [13]. Despite continuous advancements in fluoroscopic technology, cumulative radiation exposure remains a relevant concern for both patients and interventionalists [4, 14, 15].

Previous studies have reported conflicting results regarding the impact of TRA vs. TFA on radiation exposure. In diagnostic cerebral angiography and interventional cardiology, some reports have suggested higher FTs and radiation doses with TRA, especially during early adoption, whereas others found comparable or even lower doses once operators are experienced [16–18]. In the setting of EAT, available data are limited and inconsistent, and many studies either combine diagnostic and therapeutic procedures or do not adequately adjust for case complexity and operator experience. Especially, the distinction between diagnostic and therapeutic procedures is essential. While diagnostic procedures are relatively standardized, EAT is highly individualized, involving different devices and varying aneurysm configurations. As a result, it remains unclear whether the access route is an independent predictor of radiation exposure in contemporary EAT, as prior studies are heterogeneous and often do not adequately account for procedural strategy, case complexity, and operator.

To address this gap, we aimed to compare radiation exposure between TFA and TRA and to identify patient- and procedure-related factors associated with higher radiation exposure during EAT. We hypothesized that the TRA route does not result in higher radiation exposure than TFA after adjustment for patient- and procedure-related factors.

## Material and methods

The study was approved by the ethics committee of Hamburg, Germany (Chamber of Physicians, Hamburg), in accordance with the Declaration of Helsinki. Patient-informed consent was waived due to the retrospective study design. This study followed the Strengthening the Reporting of Observational Studies in Epidemiology (STROBE) reporting guideline for cohort studies.

### Study population

This retrospective single-center study included all patients who received EAT for ruptured and unruptured aneurysms at the University Medical Center Hamburg-Eppendorf during a two-year treatment period from May 2023 to April 2025.

The EAT was performed by nine experienced board-certified neuroradiologists during the routine working hours (8 am to 6 pm). The interventionalists were pseudonymized and assigned a random number known only to the first author, who was not involved in the EAT procedures. The decision for EAT was made by an interdisciplinary team consisting of neurosurgeons and neuroradiologists. The choice between TFA and TRA was left to the discretion of the treating interventionalist. Five of the nine interventionalists used both TFA and TRA, while four interventionalists used TFA exclusively. For further details on operators’ choice, see Supplement Table 2.

Treated aneurysms were located in the anterior and posterior circulation, including the following exact locations: internal carotid artery (paraophthalmic segment, posterior communicating artery (PCOM) position, carotid T), anterior communicating artery (ACOM), anterior cerebral artery (ACA), middle cerebral artery (MCA), vertebral artery (VA), basilar artery (BA), posterior cerebral artery (PCA), superior cerebellar artery (SCA) and posterior inferior cerebellar artery (PICA).

EAT included the following strategies: coiling (solely, stent-or balloon-assisted), intra-saccular devices (Woven EndoBridge, Artisse, Contour), or Flow Diverters.

### **Primary and secondary outcome parameters**

The primary outcome parameter was the dose area product (DAP, mGy·cm^2^, converted to Gy·cm^2^). FT and the number of series were collected as secondary outcome parameters to characterize case complexity; however, due to their direct correlation with DAP and their more limited clinical interpretability as standalone metrics, these variables were reported descriptively without extensive discussion, in order to avoid multicollinearity, and maintain the focus on the predefined primary endpoint. DAP, FT and the number of series were taken from the dose card, which was generated automatically for each examination and sent to our Picture Archiving and Communication System, IDS 7, Sectra. In addition, the duration of the intervention was determined, defined as the period from the groin puncture to the last recorded angiographic series. All EATs were performed using a biplane flat-panel angiographic system (Azurion 7 B20/15, Philips).

### Statistical analysis

Standard descriptive statistics were used to describe all study outcomes. Univariate distribution of metric variables was described with medians and IQRs, and categorical variables as absolute and relative frequencies. Univariable analyses were performed by linear regression analyses with DAP as the dependent variable adjusted for potential confounding variables (age, gender, access route, aneurysm location, ruptured aneurysm, treating interventionalist, treatment type). Because DAP is typically right-skewed, we performed a sensitivity analysis using log-transformed DAP (ln (DAP)) as the dependent variable; results were consistent with the primary model using untransformed DAP. In addition, residual diagnostics (histograms and residual-vs.-fitted plots) showed no major differences in linear regression assumptions, supporting the use of linear regression analysis (Supplement Table 3 and Supplement Figs. 1–3). For EAT strategies, coiling was set as the reference variable. Variables with a *p*-value < 0.10 in univariable linear regression analyses were included in the multivariable model to avoid excluding potentially relevant predictors due to limited statistical power [19]. In addition, variables that we considered clinically relevant were included in the final model. Results of the multivariable linear regression model were presented as beta-coefficients together with their 95% confidence intervals and the corresponding *p*-values. A *p*-value < 0.05 was defined as statistically significant. Statistical analyses were performed with StataMP, Version 18 (StataCorp).

## Results

A total of 209 patients with ruptured (*n* = 57; 27%) and unruptured (*n* = 157; 73%) aneurysms were included in the study. The median (IQR) age was 59 (51–68), and 53 male (25%) and 156 female (75%) patients were included. The median (IQR) BMI was 26 (23–29). In 78 patients (37%) TRA and in 131 patients (63%) TFA was chosen. 154 aneurysms (74%) were in the anterior circulation and 55 aneurysms (26%) in the posterior circulation. Most frequently, aneurysms were treated with standalone coiling (42, 20%), Woven EndoBridge (46, 22%) and Flow Diverter (68, 33%).

The overall DAP was 72.2 Gy·cm² (median; IQR, 51.6–96.7), with significantly higher radiation doses observed in the TFA group compared with the TRA group (77.4 Gy·cm², IQR 53.4–97.1 vs. 66.8 Gy·cm², IQR 50.3–94.9; *p* = 0.03). The overall number of series was 15 (median; IQR, 12–19), and the overall FT was 18 (median; IQR, 12–27). Table 1 provides a detailed overview of patients’ baseline characteristics.

**Table 1:** Patients’ baseline characteristics compared by access route. *p*-values refer to pairwise comparisons

Radiation exposure	All procedures (*n *= 209)	TRA (*n* = 78)	TFA (*n* = 131)	*p*-value
DAP (Gy·cm^2^)	72.2 (51.6–96.7)	66.8 (50.3–94.9)	77.4 (53.4–97.1)	0.03
FT (min)	18 (12–27)	14.5 (9–23)	20 (13–30)	0.001
Numbers of series (*n*)	15 (12–19)	15 (12–18)	15 (11–19)	0.708
Treatment factors				
Gender (female)	156 (75%)	55 (71%)	101 (77%)	0.290
BMI, mean ± SD	26.8 (6)	27.3 (5)	26.4 (6)	0.367
Age (years), mean ± SD	58.2 (14)	58.7 (13)	57.9 (14)	0.699
SAH (yes)	57 (27%)	10 (13%)	47 (36%)	<0.001
Circulation (anterior)	154 (74%)	60 (77%)	94 (72%)	0.412
Aneurysm details, mm median (IQR)				
• Neck	2.9 (2.2–3.9)	2.9 (2.1–3.8)	3 (2.3–3.9)	0.274
• Width	4.6 (3.5–6.5)	4.5 (3.6–6.4)	4.6 (3.3–6.7)	0.203
• Height	4.8 (3.5–6.8)	4.7 (3.5–6.6)	5.2 (3.5–7.2)	0.293
• Neck-Width ratio	1.5 (1.3–2)	1.6 (1.3–2.1)	1.5 (1.3–2)	0.905
• Aspect ratio	1.8 (1.2–2.3)	1.8 (1.3–2.4)	1.8 (1.2–2.3)	0.864
Interventionalist (Most experienced)	66 (32%)	37 (47%)	29 (22%)	< 0.001
Treatment, *n* (%)				
• Coil	42 (20%)	11 (15%)	31 (24%)	0.065
• WEB	46 (22%)	15 (19%)	24%)	
• Flow Diverter	68 (33%)	29 (37%)	29%)	
• Contour Device	24 (11%)	11 (14%)	10%)	
• Artisse Device	5 (2%)	4 (5%)	(1%)	
• Stent-assisted Coiling	20 (10%)	5 (6%)	11%)	
• Balloon-assisted Coiling	4 (2%)	3 (4%)	1 (1%)	

*TRA* transradial access, *TFA* transfemoral access, *DAP* dose area product, *FT* fluoroscopy time, *SAH* subarachnoid hemorrhage, *WEB*Woven EndoBridge

### Uni- and multivariable analyses

**Fig. 1:**
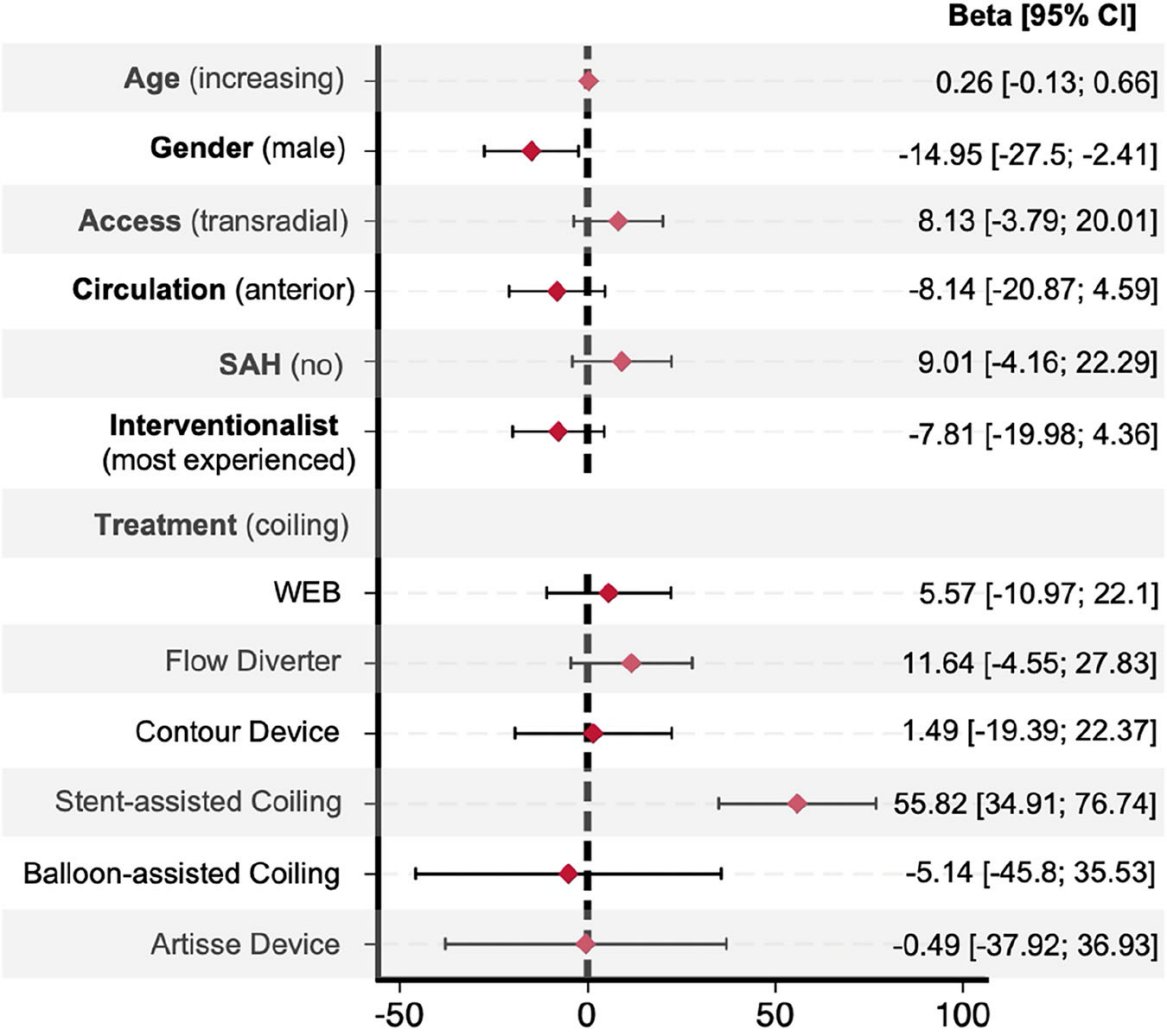
Boxplot diagram of radiation exposure by treatment type for intracranial aneurysms. Stent-assisted coiling showed significantly increased radiation exposure compared with other treatment types (*β* = 55.76 [34.55–76.97]; *p* < 0.001). DAP, dose area product; WEB, Woven EndoBridge

Univariable linear regression analyses revealed significantly increased radiation exposure for TFA (*β* = 12.9 [1.23–24.57]; *p* = 0.03) and for stent-assisted coiling (*β* = 55.76 [34.55–76.97]; *p* < 0.001) (Fig. 1). There was no significant difference in radiation exposure for gender (*β *= −10.62 [−23.66 to 2.42]; *p* = 0.11), age (*β* = 0.18 [−0.24 to 0.59]; *p* = 0.398), the treating interventionalist (*β* = 8.74 [−3.48 to 20.96]; *p* = 0.16), SAH (*β* = 9.71 [−3.04 to 22.46]; *p* = 0.135) and the circulation site (*β* = −3.85 [−16.8 to 9.09]; *p* = 0.558).

In the multivariable linear regression model, patient characteristics and treatment details were included and tested for significant associations with radiation exposure.

**Fig. 2:**
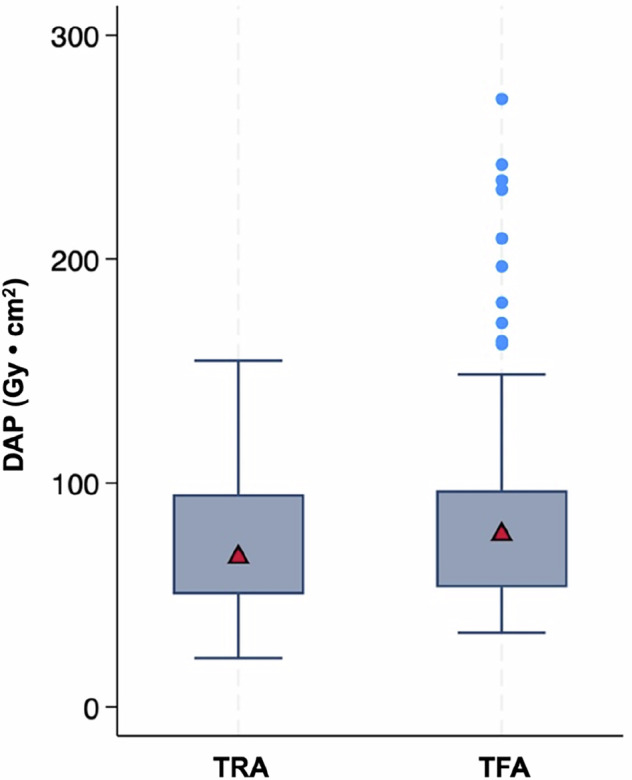
Forest plot of multivariable linear regression analysis for radiation exposure. Female gender (*β *= −14.9 [−27.5 to −2.4]; *p *= 0.02) and stent-assisted coiling (*β *= 55.8 [34.9 to 76.7]; *p *< 0.001) were independently associated with higher radiation exposure. Forest plot showing regression coefficients (beta) with 95% confidence intervals for baseline variables and treatment strategies in relation to DAP. Points represent point estimates and horizontal lines indicate 95% CIs; the vertical dashed line denotes the null effect. Point estimates above one favor higher DAP values. SAH, subarachnoid hemorrhage; WEB, Woven EndoBridge

Testing for patient characteristics showed that female gender was independently associated with radiation exposure, receiving significantly higher radiation doses compared to male patients (*β *= −14.9 [−27.5 to −2.4]; *p* = 0.02). Further patient characteristics, including age, access route (TRA vs. TFA), circulation (anterior vs. posterior), SAH (yes vs. no) and treating interventionalist (most experienced vs. others) were not independently associated with DAP in the adjusted model. Of the different procedural techniques, stent-assisted coiling was associated with a significantly higher radiation exposure (*β* = 55.8 [34.9–76.7]; *p* < 0.001). All other devices showed no statistically significant difference in DAP (Fig. 2 and Supplement Table 1).

Sensitivity analyses using log-transformed DAP (ln (DAP)) yielded results consistent with the primary analyses, with unchanged main associations (Supplement Table 3 and Supplement Figs. 1–3).

## Discussion

This study investigating the impact of arterial access routes on radiation exposure during EAT revealed the following main findings: (1) no significant difference in the DAP was observed between TRA and TFA, indicating comparable levels of radiation exposure for both approaches; (2) radiation exposure was significantly influenced by the endovascular treatment strategy with stent-assisted coiling showing an association towards higher DAP, likely due to more complex aneurysm configurations.

Interventional cardiology has established the TRA route as the primary approach for coronary angiography and percutaneous coronary interventions with reduced complication rates and a high safety profile [16, 20]. In the past years, TRA has also been increasingly used in neurointerventional procedures [17]. However, challenges associated with TRA, e.g., lack of a variety of dedicated catheters, slower learning curve, and artery navigation, have been a concern for treatment duration and subsequent higher radiation doses [3]. Recent studies on endovascular treatment of intracranial aneurysms reported a radiation dose ranging between 94 and 179 Gy·cm^2^ [18, 21–23] for endovascular treatment of intracranial aneurysms. In our study, the radiation dose was slightly below this range with a median DAP (IQR) of 72 Gy·cm^2^, and by that below the recommended reference level of the Federal Office for Radiation Protection [24]. Other radiation parameters, such as FT and the number of series, were collected and reported descriptively, but were not included in the primary modeling due to their strong correlation with DAP and the risk of multicollinearity.

**Fig. 3:**
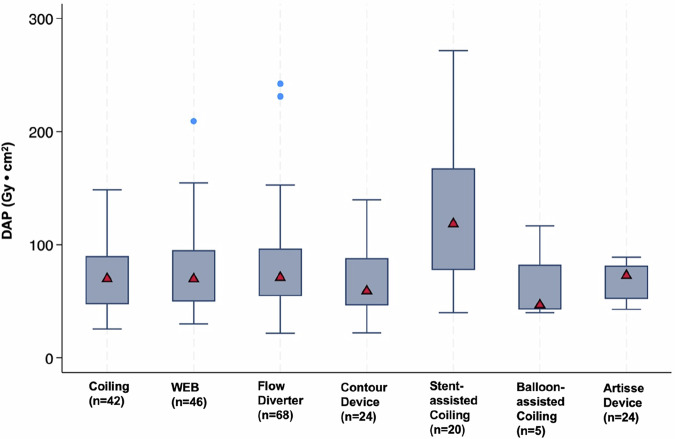
Boxplot of radiation exposure by access route. Median DAP was significantly higher in the TFA group than in the TRA group (77.4 Gy·cm², IQR 53.4– 97.1 vs. 66.8 Gy·cm², IQR 50.3–94.9; *p* = 0.03)

A recent retrospective study found significantly higher radiation exposure in TRA compared to TFA during cerebral diagnostic angiography [25], while a meta-analysis of EAT showed no consistent advantage of TRA in terms of procedure time, resulting in increased radiation exposure [6]. Comparing radiation exposure by access route in our cohort, we could not corroborate the aforementioned concerns of higher radiation doses in TRA. Although univariable analyses indicated significantly higher DAP in procedures with TFA access, this association did not remain significant after adjusting for potential confounders in a multivariable model, showing similar rates of DAP for TRA and TFA (Fig. 3). In our cohort, TFA was used more frequently in patients with ruptured aneurysms and subsequent SAH (TFA 36% vs. TRA 13%; *p* < .001), which are typically associated with higher procedural complexity and increased radiation exposure. Moreover, the most experienced operator was disproportionately represented in the TRA group (TRA 47% vs. TFA 22%; *p* < .001), whereas TFA procedures were more often performed by less experienced operators. Both rupture status and operator experience are plausible determinants of radiation exposure and may substantially influence DAP independent of access route. The higher radiation exposure observed for TFA in univariable analysis, therefore, likely reflects confounding by case mix and operator allocation rather than a true effect of the access route itself. After adjustment for these factors in the multivariable model, the association between access route and DAP was attenuated and no longer statistically significant, supporting this interpretation. Because the choice of access was left to the discretion of the operator, residual confounding by case complexity cannot be fully excluded despite multivariable adjustment, and our access-related findings should therefore be interpreted carefully. Consequently, the TRA route may be considered in rare stroke cases where minimizing patient radiation exposure is particularly important, such as in pregnant patients, due to improved controllability and a reduced risk of fetal radiation exposure [26].

Procedural complexity is known to increase radiation exposure due to longer intervention times. In our analysis, stent-assisted coiling, generally used for more complex, wide-necked aneurysms, was independently associated with higher DAP, showing a significant increase of 55.8 Gy·cm² compared with coiling, after adjustment for key covariates such as the interventionalist’s experience and the access route [27, 28]. Corroborating this observation, Cheung et al similarly reported higher DAP for stent-assisted compared to standalone coiling procedures [21]. These results highlight the role of the complexity of aneurysm configuration as a key determinant of radiation exposure, independent of access route and interventionalist’s experience.

Our analysis showed that female patients had a significantly higher DAP after multivariable adjustment, with a mean difference of 14.9 Gy·cm^2^ compared to male patients. This finding is in contrast with a recent multicenter study by Ihn et al, in which male patients undergoing EAT received significantly higher radiation doses than female patients [18]. This discrepancy may be explained by differences in patient populations, with our study focusing solely on EAT, along with variations in aneurysm characteristics or treatment complexity across cohorts [29]. In addition, several biological and procedural mechanisms could contribute to sex-related differences in radiation exposure, including differences in body habitus, vessel size and tortuosity, or aneurysm morphology. Fluoroscopy systems use automatic exposure control, which adjusts radiation output according to patient attenuation; subtle differences in soft tissue distribution may therefore influence DAP [30]. However, quantitative measures of vascular tortuosity, body habitus beyond BMI, and soft tissue attenuation were not systematically collected in our cohort, which precludes a definitive explanation of the observed sex difference in DAP. Therefore, this finding should be interpreted as hypothesis-generating and warrants confirmation in larger, prospective studies. Further studies are needed to identify patient-and gender specific factors influencing radiation exposure in EAT procedures.

This study has several limitations that should be considered when interpreting the results: 1) due to its retrospective design, there is an inherent risk of selection bias and limited control over confounding variables; 2) the analysis was conducted at a single-center, which may limit the generalizability of the findings to other institutions with different interventional protocols, technical equipment, or operator experience; 3) the choice of access route was not standardized and was left to discretion of the treating interventionalist, which may have introduced allocation bias; and 4) quantitative measures of vascular tortuosity were not systematically recorded, which limited our ability to explore potential mechanisms underlying the observed sex difference in DAP.

## Conclusion

Radiation exposure did not differ significantly between TRA and TFA in the endovascular treatment of intracranial aneurysms. Thus, the choice of access route should not influence treatment strategy when considering radiation doses of the procedure. Higher radiation doses were independently associated with stent-assisted coiling, most likely reflecting the increased procedural complexity of treating complex, wide-necked aneurysms. Further prospective, multicenter data are needed to confirm these results.

## Supplementary information

Supplementary information

## Abbreviations

DAP: Dose area product
EAT: Endovascular aneurysm treatment
FT: Fluoroscopy time
TFA: Transfemoral approach
TRA: Transradial approach
