# Supplementary material for: Radiation exposure in transradial versus transfemoral access in endovascular treatment of intracranial aneurysms
Source: Eur Radiol. 2026 May 4;36(9):7305–12. doi: 10.1007/s00330-026-12603-7 (PMC13451452; doi:10.1007/s00330-026-12603-7)
Supplement: Supplementary file 1 [file 330_2026_12603_MOESM1_ESM.pdf]

Radiation Exposure in Transradial versus Transfemoral Access in Endovascular Treatment of Intracranial Aneurysms
ELECTRONIC SUPPLEMENTARY MATERIAL

Supplement Figure 1: Histogram and kernel density plot of dose area product (DAP), demonstrating a right-skewed distribution of radiation exposure values.

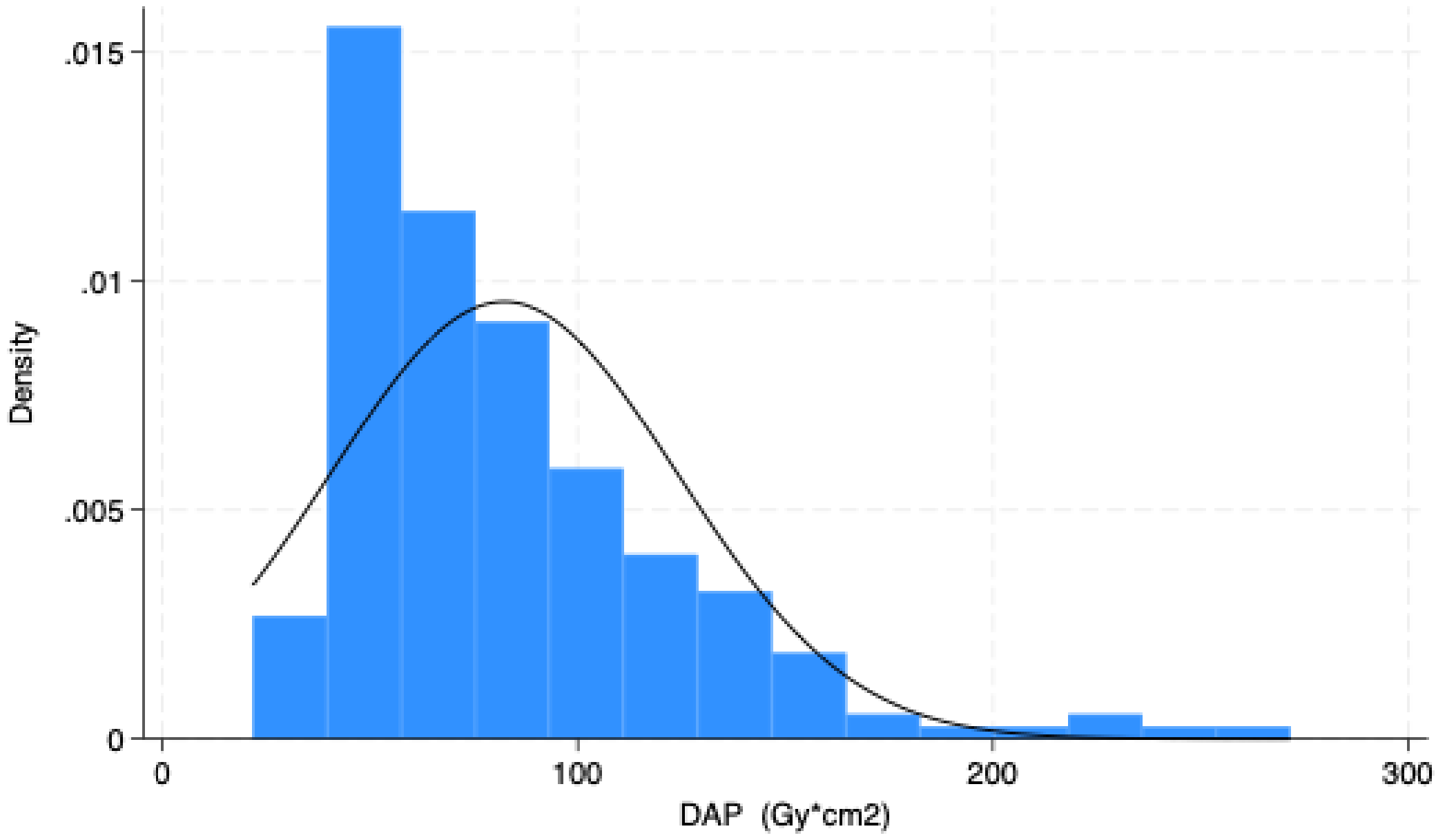

Supplement Figure 2: Histogram and kernel density plot of logarithmically transformed dose area product (ln[DAP]), showing a more symmetric distribution compared with untransformed DAP.

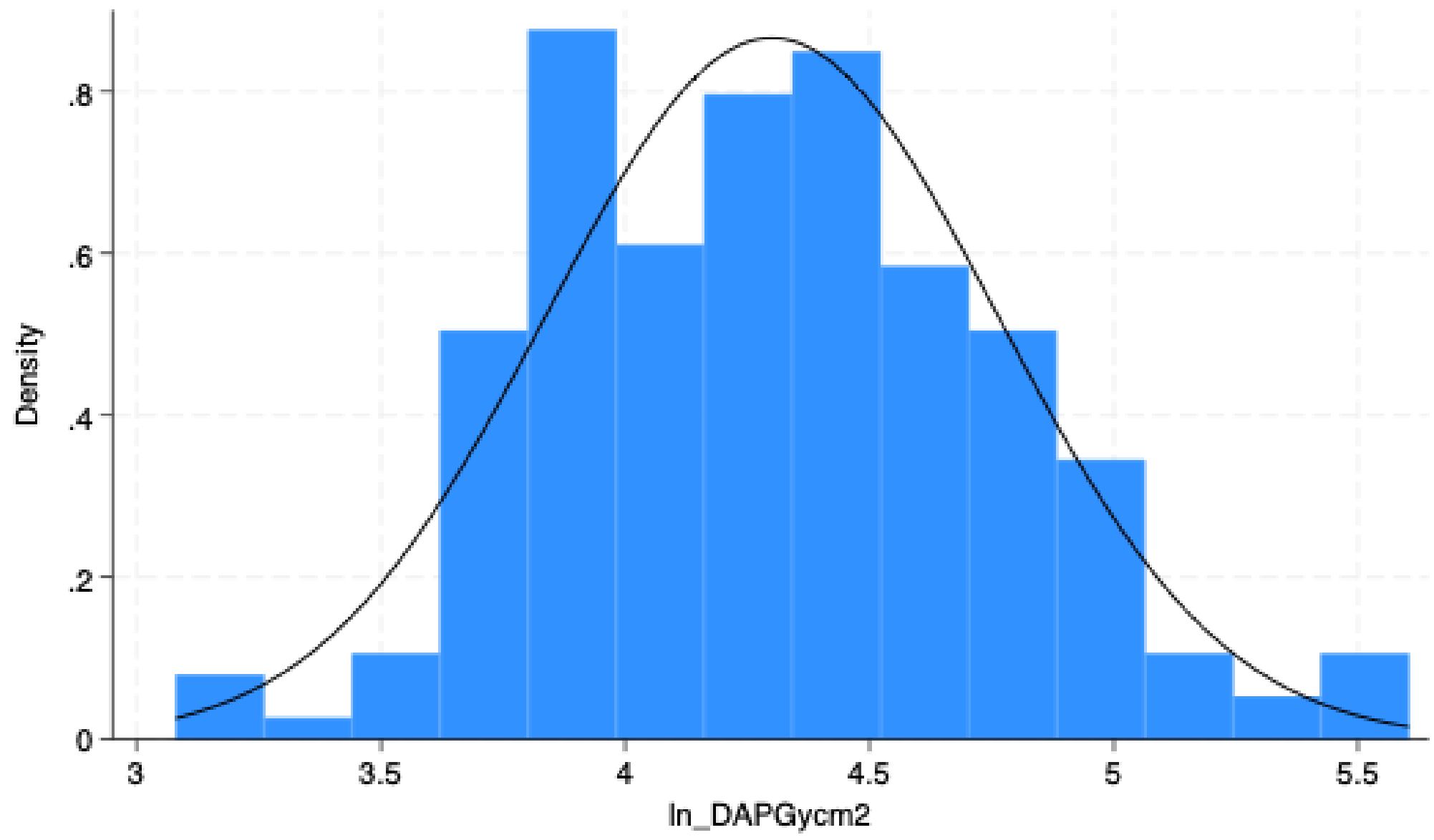

Supplement Figure 3: Multivariable linear regression analysis with logarithmically transformed dose area product (ln[DAP]) as the dependent variable. Regression coefficients represent relative changes in radiation exposure. Results are shown to assess the robustness of the primary analysis using untransformed DAP.

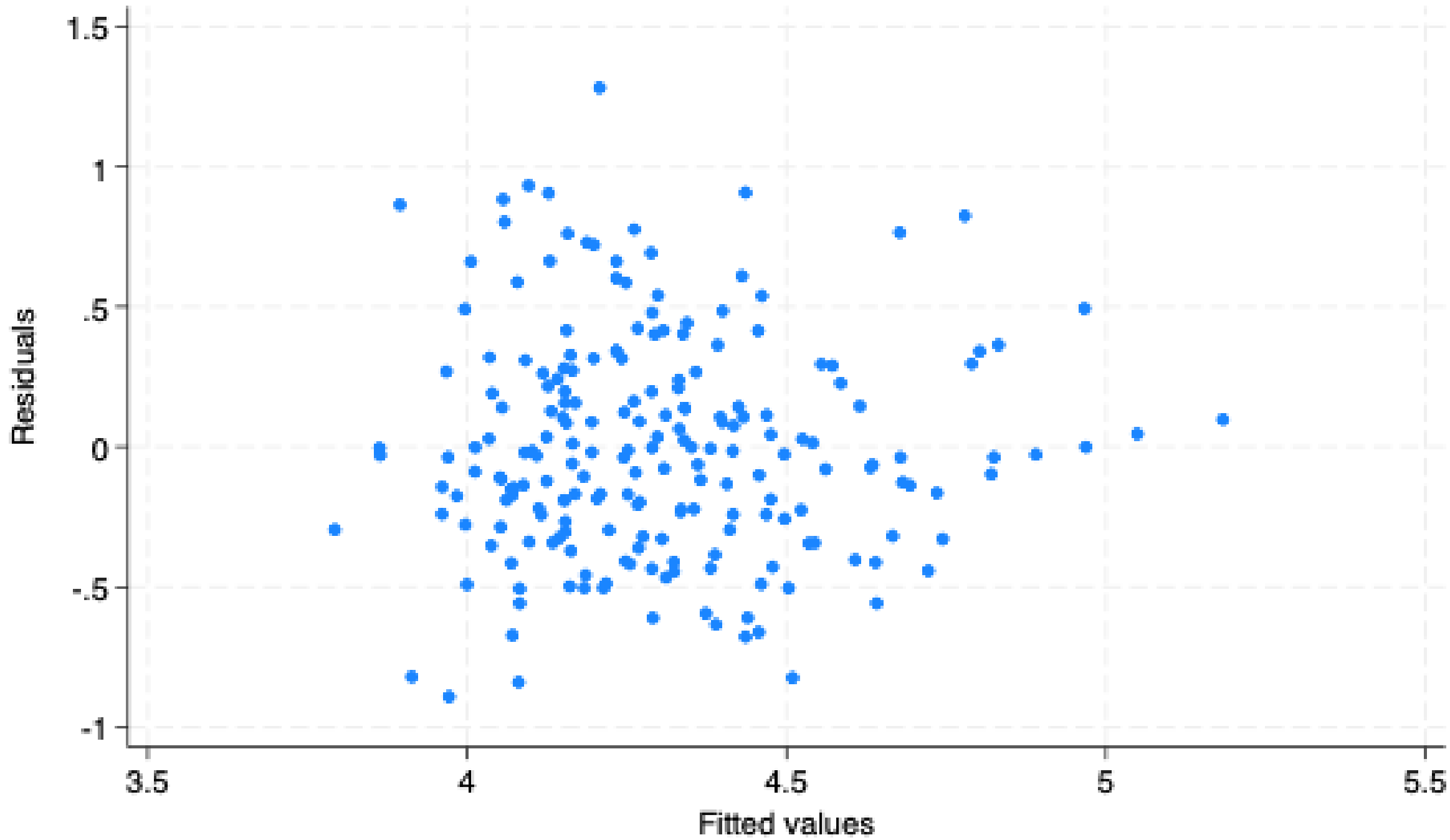

Supplement Table 1: Univariable and multivariable linear regression analyses for radiation exposure

Baseline variable	Univariable β coefficient (95% CI)	p-value	Multivariable β coefficient (95% CI)	p-value
▪ Age (increasing)	0.18 (-0.24-0.59)	0.398	0.26 (-0.13-0.66)	0.185
▪ Gender (male)	-10.62 (-23.66-2.42)	0.11	-14.95 (-27.5- -2.41)	**0.02**
▪ Access (transradial)	12.9 (1.23-24.57)	**0.03**	8.13 (-3.79-20.01)	0.18
▪ Circulation (anterior)	-3.85 (-16.8-9.09)	0.558	-8.14 (-20.87-4.59)	0.209
▪ SAH (no)	9.71 (-3.04-22.46)	0.135	9.01 (-4.16-22.29)	0.178
▪ Interventionalist (most experienced)	8.74 (-3.48-20.96)	0.16	-7.81 (-19.98-4.36)	0.207
▪ Treatment (coiling)				
- WEB	5.72 (-11.02-22.46)	0.501	5.57 (-10.97-22.1)	0.508
- Flow Diverter	8.12 (-7.31-23.55)	0.301	11.64 (-4.55-27.83)	0.158
- Contour device	2.56 (-16.95-22.06)	0.796	1.49 (-19.39-22.37)	0.888
- Stent-assisted coiling	55.76 (34.55-76.97)	**<.001**	55.82 (34.91-76.74)	**<.001**
- Balloon-assisted coiling	-10.43 (-51.03-30.18)	0.613	-5.14 (-45.8-35.53)	0.804
- Artisse device	-5.35 (-42.08-31.38)	0.774	-0.49 (-37.92-36.93)	0.979

Supplement Table 2: Operators choice of access and TRA/TFA ratio

Operator	TRA (%)	TFA (%)	Total	TRA/TFA Ratio
**1**	37 (56)	29 (44)	66	0.56/1
**2**	0 (0)	22 (100)	22	0/1
**3**	1 (8)	11 (92)	12	0.08/1
**4**	2 (17)	10 (83)	12	0.17/1
**5**	19 (56)	15 (44)	34	0.56/1
**6**	19 (37)	33 (63)	52	0.37/1
**7**	0 (0)	9 (100)	9	0/1
**8**	0 (0)	1 (100)	1	0/1
**9**	0 (0)	1 (100)	1	0/1

Legend:
TRA = Transradial access
TFA= Transfemoral access

Supplement Table 3: Multivariable linear regression analysis with logarithmically transformed dose area product (ln[DAP]) as the dependent variable. Regression coefficients represent relative changes in radiation exposure. Results are shown to assess the robustness of the primary analysis using untransformed DAP

Baseline variable	Coefficient (β)	p-value	95% Confidence interval
▪ Age (per year)	0.003	0.125	-0.001-0.008
▪ Female sex	-0.19	0.007	-0.33- -0.05
▪ Access (transradial)	0.068	0.332	0.07-0.21
▪ SAH (no)	0.086	0.241	-0.06-0.23
▪ Circulation (anterior)	-0.153	0.034	-0.29- -0.01
▪ Interventionalist (most experienced)	0.14	0.367	-0.17-0.45
▪ Treatment (coiling)			
- WEB	0.037	0.691	-0.15-0.22
- Flow Diverter	0.131	0.144	-0.05-0.31
- Contour device	0.006	0.961	-0.22-0.23
- Stent-assisted coiling	0.574	<.001	0.35-0.8
- Balloon-assisted coiling	-0.212	0.346	-0.66-0.23
- Artisse device	-0.048	0.817	-0.45-0.36
